# Bleeding disorders in implant dentistry: a narrative review and a treatment guide

**DOI:** 10.1186/s40729-022-00418-2

**Published:** 2022-04-16

**Authors:** Paul Römer, Diana Heimes, Andreas Pabst, Philipp Becker, Daniel G. E. Thiem, Peer W. Kämmerer

**Affiliations:** 1 Department of Oral- and Maxillofacial Surgery, University Medical Center Mainz, Augustusplatz 2, 55131 Mainz, Germany; 2 Department of Oral and Maxillofacial Surgery, Federal Armed Forces Hospital, Rübenacher Straße 170, 56072 Koblenz, Germany

**Keywords:** Bleeding disorder, Antiplatelet drug, Anticoagulant, Hemophilia, Factor deficiency, von Willebrand disease

## Abstract

**Purpose:**

Considering a high prevalence of congenital and especially acquired bleeding disorders, their heterogeneity and the multitude of possible treatments strategies, a review of the scientific data on this topic is needed to implement a treatment guide for healthcare professionals.

**Methods:**

A selective literature review was performed via PubMed for articles describing oral surgery / dental implant procedures in patients with congenital and acquired bleeding disorders. Out of the existing literature, potential treatment algorithms were extrapolated.

**Results:**

In order to assess the susceptibility to bleeding, risk stratification can be used for both congenital and acquired coagulation disorders. This risk stratification, together with an appropriate therapeutic pathway, allows for an adequate and individualized therapy for each patient. A central point is the close interdisciplinary cooperation with specialists. In addition to the discontinuation or replacement of existing treatment modalities, local hemostyptic measures are of primary importance. If local measures are not sufficient, systemically administered substances such as desmopressin and blood products have to be used.

**Conclusions:**

Despite the limited evidence, a treatment guide could be developed by means of this narrative review to improve safety for patients and practitioners. Prospective randomized controlled trials are needed to allow the implementation of official evidence-based guidelines.

## Background

Every dental surgical intervention involves a certain risk of postoperative bleeding. In dental implantology, the treatment may involve moderately to highly invasive surgical procedures. From “simple” tooth extractions with immediate implant placement to complex multi-stage procedures involving augmentations and/or sinus lift procedures, the patient-specific bleeding risk must always be correctly assessed, and sufficient precautions have to be implemented. The incidence of postoperative bleeding after dental surgical procedures in healthy individuals is approximately 0.2–3.3%, whereas in patients with coagulation disorders, such complications have been reported to be significantly more common at 8.6–32.1% [1]. Special attention and preparation is therefore required for those patients who are exposed to an increased risk of bleeding.

The diseases in question can be divided into congenital and acquired blood coagulation disorders. Congenital coagulation disorders include the von Willebrand–Jürgens syndrome, which affects up to one percent of the population, and hemophilia A and B, subtypes of which are mild, moderate, and severe, depending on the residual factor activity [2, 3]. Acquired blood coagulation disorders are often concomitant symptoms of liver, kidney, and bone marrow diseases, which, depending on their severity and genesis, require particular caution, especially when invasive surgical procedures are imminent. Furthermore, in addition to congenital and acquired blood coagulation disorders, knowledge of drugs that influence blood coagulation is becoming increasingly important. Approximately, one percent of the German population is already being treated with oral anticoagulants [4]. It is important to know the patient’s general diseases and to be able to distinguish between different forms of oral anticoagulation drugs and their half-life period. The purpose of this narrative review is to provide an overview of the current literature and, based on these results, to present recommendations for the treatment of patients with those very bleeding disorders.

## Materials and methods

A selective literature review was performed via PubMed for articles describing oral surgery/dental implant procedures in patients with congenital and acquired bleeding disorders. Out of the existing literature, potential treatment algorithms were extrapolated. Randomized controlled trials (RCTs), prospective studies, observational studies, reviews, and retrospective studies were preferred; case reports were used where no further data sources were available due to the scarcity of data.

## Literature review on bleeding disorders in dentistry

### Congenital bleeding disorders

Congenital bleeding disorders are hereditary diseases which are clinically characterized by an increased tendency to bleeding (spontaneous and/or after trauma/invasive procedures) due to genetic abnormalities concerning quantitative and/or qualitative components of the coagulation system.

Extent and severity of bleeding events depend on such factors as severity of the corresponding pathology, local and systemic patient factors and type of intervention. If the treatment is not sufficiently adapted to the patient’s needs, extended hospitalization time or psychological traumata may occur. A review of dental interventions on patients with mild blood clotting disorders also showed that unnecessary over-treatment was performed in 59% of the cases [5].

### von Willebrand syndrome

Up to one percent of the population suffers from von Willebrand disease (VWD), a usually autosomal dominant inherited quantitative or functional deficit of the factor VIII carrier protein, affecting the von Willebrand factor (VWF). However, the prevalence of clinically relevant VWD is significantly lower.

VWD results from an either quantitative (type 1 or 3) or qualitative (type 2) deficiency of VWF in the patients’ blood plasma (Table 1). Affected patients can suffer from varying degrees of bleeding during and after invasive procedures. Blombäck et al. reported of a high complication rate in patients with unknown VWD at the time of surgical intervention (67%), while corresponding complications only occurred in 6.7% of patients who were aware to have the disease and therefore had been treated systemically [6].

**Table 1:** Different types of von Willebrand disease (VWD)

Type 1		Quantitative deficiency of VWF	80% of patients
Type 2	A	Qualitative deficiency of VWF with reduced binding ability of VWF to platelets and collagen and reduced high molecular monomers	15–20% of patients
	B	Qualitative deficiency of VWF with increased affinity for platelet-derived GPIb-receptor and reduced high molecular monomers	
	M	Reduced affinity to platelets with a functional defect in primary hemostasis and decreased ratio of ristocetin cofactor activity to VWF antigen	
	N	Reduced affinity for factor VIII	
Type 3		Complete absence or severe reduction of VWF	0.5–1.5% of patients

The VWD is divided into three types (with four subtypes of type 2). In type 1 and 3, von Willebrand factor (VWF) is quantitatively decreased. In type 2 (A,B,M,N) the VWF is qualitatively defective

Desmopressin (usually 0.3 µg/kg bodyweight [BW] at least 1 h before intervention [7]) is used systemically in patients with type-1-vWD and available residual concentration of factor VIII and vWF-concentrations of > 10 IE/dl. Patients who are not responding to desmopressin, as well as patients with other types of vWD can be treated with vWF-containing factor VIII concentrates [8–10]. De Padua et al. investigated whether patient-specific therapy protocols, based on type and severity of the vWD and the invasiveness of the intervention, could prevent perioperative bleeding. Local hemostatic measures (gelatin cone, fibrin glue, adapting sutures, compression, application of tranexamic acid, cooling) were applied in all procedures. Overall, no peri- or post-operative bleeding events could be observed [10].

In case of type 1, the majority of affected patients only shows slightly decreased vWF-plasma concentrations and typically no hemorrhagic diathesis. Anyhow, it could be shown that in this cohort, despite minor vWF-plasma reduction, there is a correlation to a clinically increased bleeding phenotype. Potential reasons for this can be a reduction in vWF synthesis and/or in its consecutive secretion, overall, however, there is not yet enough evidence here [11, 12]. Doherty et al. examined a total of 60 patients with slightly decreased vWF-plasma levels in combination with a known history of bleeding phenotype, in which at least one elective procedure had been performed before “low vWF bleeding phenotype” was diagnosed. Retrospectively, in 62.5% of the cases (30/48) bleeding events could be observed and further measures, such as compression, sutures or transfusions became necessary in 43.8% of the cases (23/48). As part of a prospective study, 40 dental procedures (mostly single tooth extractions) were performed under local hemostatic measures in addition to systemic administration of tranexamic acid (1 g/3 × days) in 25% of the cases and desmopressin (0.3 μg/kg BW) in 72.5% of the cases. As a result, only 10% of the treated patients showed post-interventional bleeding events [13].

*Conclusion* The peri- and post-interventional care of patients with vWD for dental implantology requires close interdisciplinary collaboration, especially with hematologic disciplines. Desmopressin and factor concentrates are used depending on the type of vWD to significantly reduce the risk of bleeding events. The elective management of patients with slightly decreased vWF and “bleeding phenotype” is a major clinical challenge with a current lack of evidence and uncertain definition. Intravenous administration of desmopressin is a safe and effective treatment for elective dental procedures in those patients. However, especially in elderly patients with comorbidities, contraindications to administration of desmopressin are frequent.

### Hemophilia A and B

Deficiencies of clotting factor VIII (hemophilia A) and factor IX (hemophilia B) occur with a prevalence of approximately 1:10–20,000 and 1:30–60,000, respectively [2, 3], of which about 2/3 of the affected hemophilia patients indicate a positive family disposition, while other clinical pathologies are more likely to be spontaneous new mutations.

Hemophilia A and B are divided into three degrees of severity, depending on the quantitative deficit of remaining factor activity [14]. Residual factor activities of > 25% are required for a generally normal hemostasis while most of the patients feature levels below 5% [15]:

- mild hemophilia: factor activity 5–40%,
- moderate hemophilia: factor activity 1–5% and
- severe hemophilia: factor activity < 1% [16, 17].

Infusion of factor preparations is considered the gold standard to counteract bleeding events, however, frequent and weight-adapted doses are required due to short half-life periods [17]. The main complication of substitution therapy with a life-long risk of 5–40% is the occurrence of antibodies against factor VII or IX in the recipient’s blood, which are known as “inhibitors” that neutralize the effect of the factor concentrate and thus making the treatment more difficult. Therefore, patients with mild hemophilia are usually only treated with concentrate of the analogous factor in the case of a surgical intervention, an accident, or a serious injury [18].

**Fig. 1:**
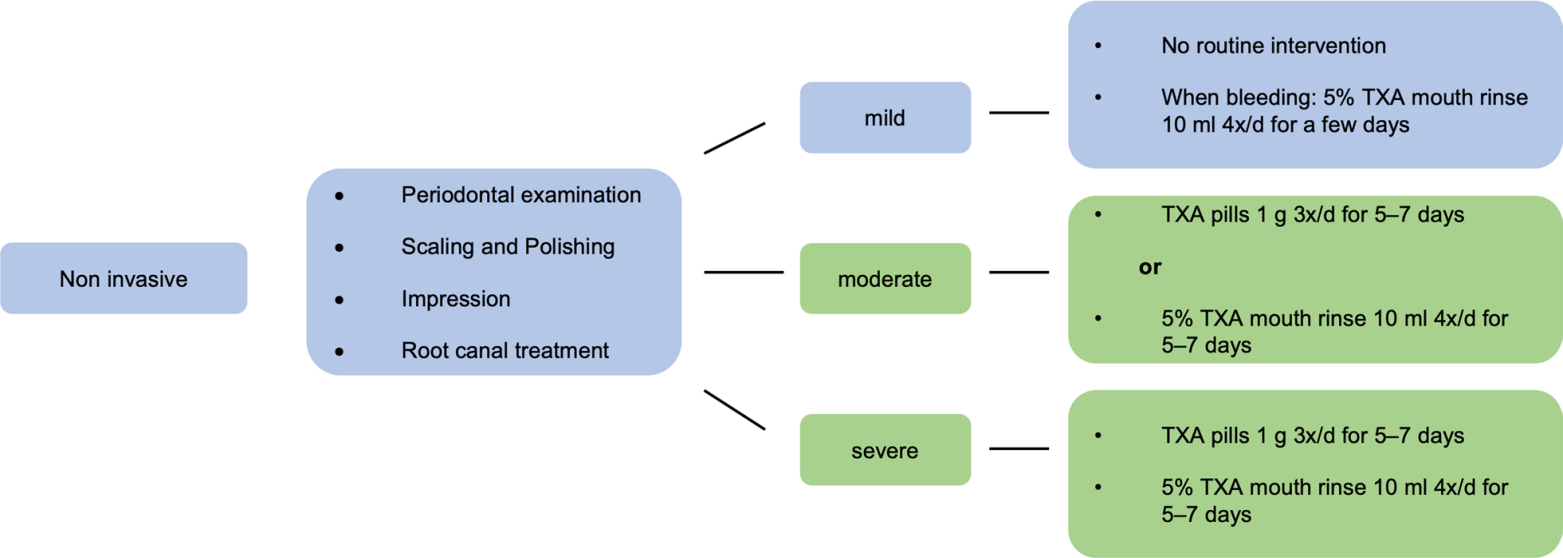
Exemplary treatment scheme for noninvasive dental treatments. No risk (blue) and low risk (green) (acc. to [2]). TXA: tranexamic acid

**Fig. 2:**
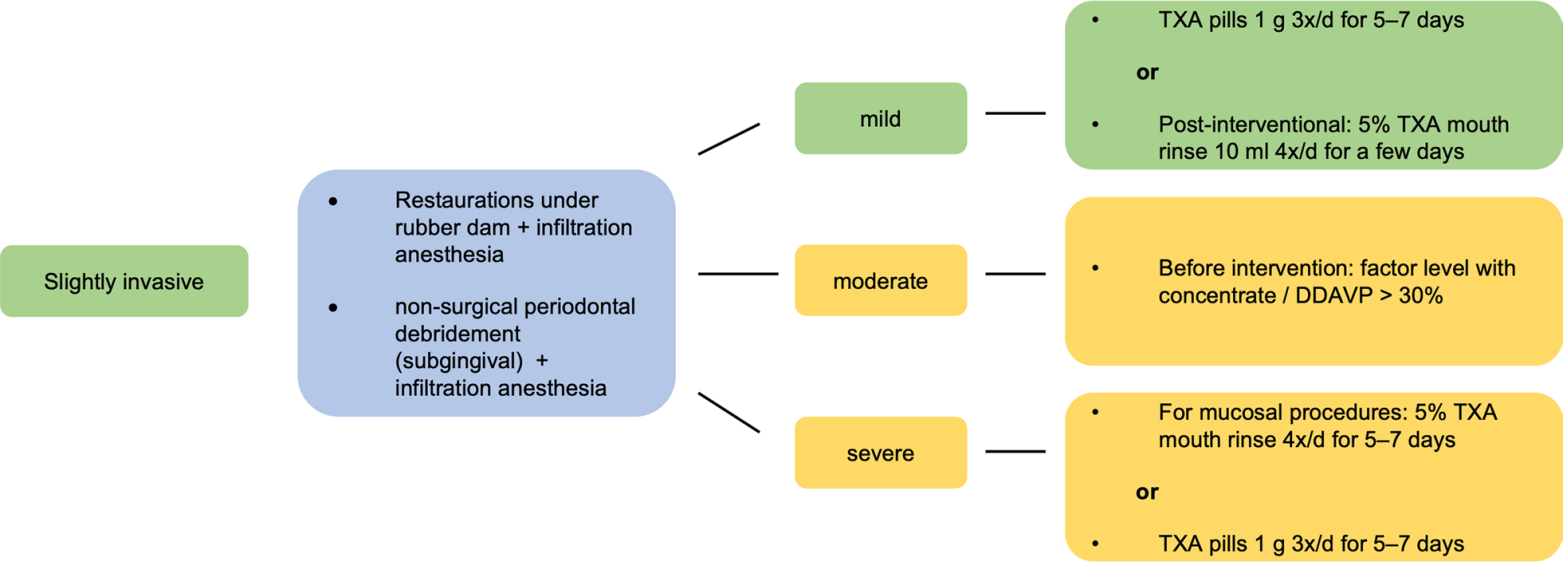
Exemplary treatment scheme for slightly invasive dental treatments. Low risk (green) and moderate risk (yellow) (acc. to [2]). *TXA* tranexamic acid, *DDAVP* desmopressin

**Fig. 3:**
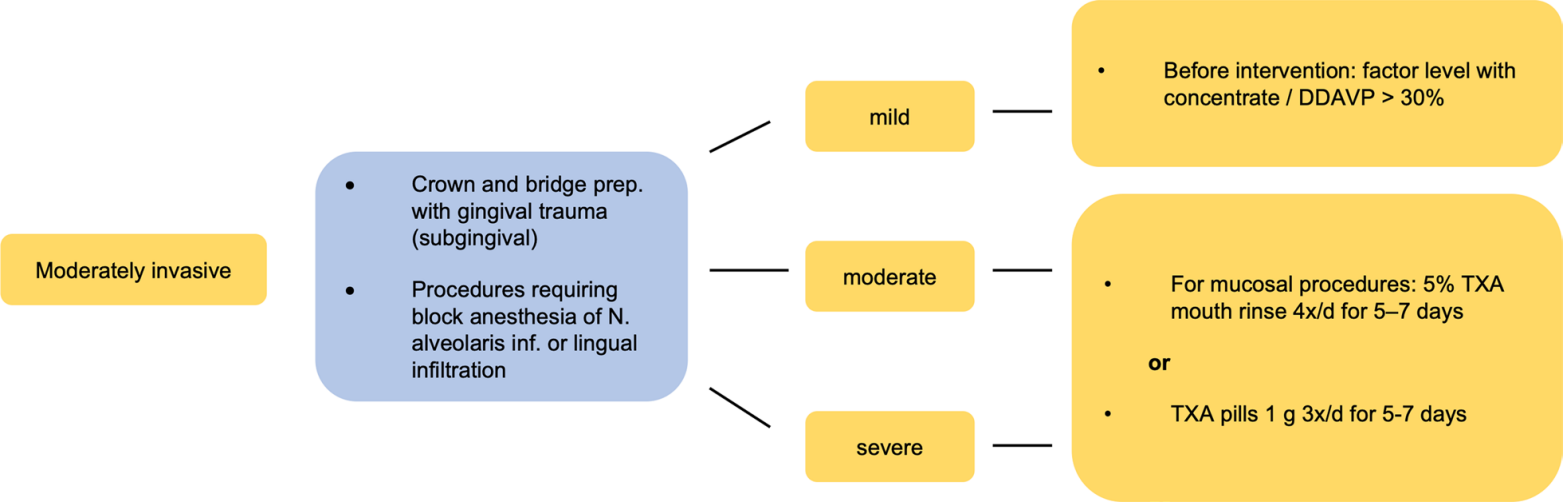
Exemplary treatment scheme for moderate-risk dental treatments. Moderate risk (yellow) (acc. to [2]). TXA: tranexamic acid; DDAVP: Desmopressin

**Fig. 4:**
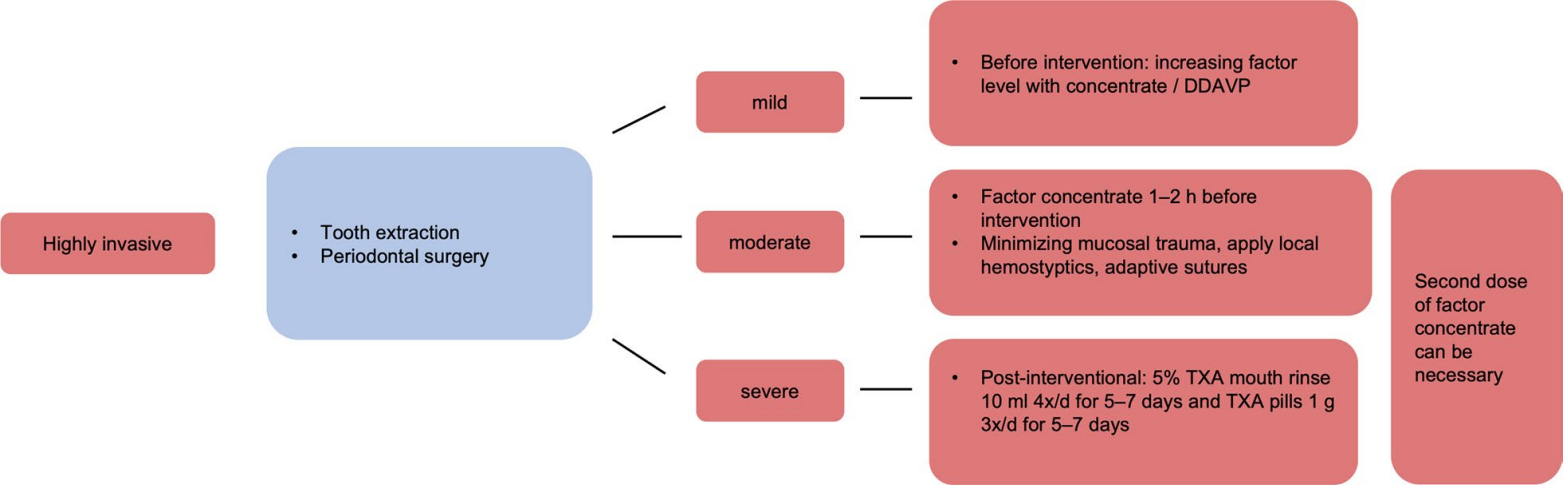
Exemplary treatment scheme for highly invasive dental treatments. High risk (red) (acc. to [2]). TXA: tranexamic acid; DDAVP: Desmopressin

Hence there is a need to carefully consider the use of factor substitution, which is why, for example, in procedures such as regular dental examination, supragingival scaling, root canal treatments, simple fissure sealing and restorative dental treatments without the need for conduction a nerve block, no extended measures need to be prepared. In contrast, tooth extraction, implant insertion, or treatment of advanced periodontal diseases tend to require substitution of the missing factors and/or additional local hemostatic measures (Figs. 1, 2, 3, 4) [14, 19, 20]. Patients who nonetheless have developed inhibitory antibodies are subsequently primarily treated with either recombinant activated factor VII or activated prothrombin complex concentrates derived from blood plasma [21].

As an alternative to factor concentrates, desmopressin can be used in mild cases. However, the evidence for the exact protocols is limited and there is little or no consensus regarding the ideal level and duration of appropriate therapies for preventing bleeding in oral surgery. The use of local hemostyptics alone does not seem to be sufficient, especially in cases of moderate or severe hemophilia [14], making additional peri-interventional adjuvants necessary during treatment.

In a review article, Bajkin und Dougall examined hematological preparation protocols (factor concentrates pre- and/or post-operatively, Desmopressin, systemic antifibrinolytic drugs 5–10 days) and calculated a rebleeding rate of 3.9–27.8%. No evidence could be found whether hemophilia patients should be treated as an outpatient or inpatient, although severe bleeding complications were only reported in few cases [22, 23]. Laino et al. attempted to extract guidelines for the treatment of hemophilia patients in the context of oral and maxillofacial surgery form existing literature (7 articles) [24]. The authors point out that ingestion of non-steroidal anti-inflammatory drugs (NSAIDs) is considered to be contraindicated due to their negative effect on platelet aggregation, while paracetamol and codeine are more recommended [24], although no corresponding studies in dental or oral surgery are available. Patients with mild-to-moderate hemophilia benefit from less invasive forms of local anesthesia, such as infiltration anesthesia or need for factor substitution before more invasive forms, but evidence is limited [24].

*Conclusion* Overall, only poor-to-moderate evidence exists for oral surgical treatment of hemophilia patients. The relevant guidelines are based primarily on empirical data obtained from clinical experience of centers specialized in the treatment of hemophilia. A recent review [22] reported similar postoperative bleeding rates in patients with hemophilia after dental extractions who had received factor concentrates either both preoperatively and postoperatively or only before intervention.

### Congenital bleeding disorders and local anesthesia

Dougall et al. conducted a prospective study (71 patients, no control group) with patients deficient in factors VIII, IV and XI (mild to severe hemophilia) who required dental procedures under local anesthesia [25]. In these patients, buccal infiltrations were performed without further hematological treatment. The authors observed no bleeding or hematoma at the injection site concluded that local anesthetics can be administered safely via buccal injection in patients with mild, moderate and severe hemophilia without additional factor coverage [25]. This is analogous to the World Federation of Hemophilia guidelines, which describe infiltration anesthesia in people with hemophilia as safe and without the need for factor substitution [19]. In contrast, factor substitution is recommended before block anesthesia because of the increased potential for hemorrhage with the risk of intramuscular hematoma and thus, at worst, potential airway obstruction. Nonetheless, it must be noted that this guideline was consensus-based due to the lack of supporting clinical data and the absence of reports of life-threatening bleeding events after such block anesthesia. For example, Hewson et al. reported 41 block anesthesias in patients with congenital coagulation disorders without evidence for complication [26].

### Congenital bleeding disorders and topical hemostyptics

In the dental and oral surgical treatment of patients with congenital coagulation disorders, the recommendations for bleeding prophylaxis are based on the four pillars:

1. Compensation of factor deficiency by appropriate concentrates
2. Desmopressin administration in a suitable collective
3. Systemic administration of antifibrinolytics
4. Use of topical antifibrinolytics and topical hemostyptics.

The choice for a locally effective topical hemostyptic can potentially limit the need for systemic substitutions which may be relevant for clinical as well as economic and social reasons [26]. The establishment of a stable blood clot usually provides adequate hemostasis as a “natural dressing”.

The management of peri-interventional bleeding in patients with vWD using local measures such as antifibrinolytics was systemically investigated by van Galen et al., but no corresponding randomized trials could be identified, thus no higher-grade evidence is available here [27]. A recent Cochrane-Review failed to demonstrate any efficacy for oral surgery in hemophilia patients due to a limited number of appropriate studies [28], although a beneficial effect of these drugs in preventing postoperative bleeding could be proven [27].

### A treatment tool for congenital bleeding disorders

Using the Dental Bleeding Risk Assessment and Treatment Tool (DeBRATT), Rasaratnam et al. stratified patients according to the invasiveness of the dental procedure (noninvasive, mild, moderate, and highly invasive) and the severity of the coagulopathy (mild, moderate, severe). The bleeding risk groups were divided into four groups:

1. No risk (blue)
2. Low risk (green)
3. Moderate risk (yellow)
4. High risk (red).

Rasaratnam et al.’s analysis of 200 dental procedures performed in 30 patients with congenital coagulation disorders following this protocol only reported one case (0.5%) of postoperative bleeding. Yet, because this was a retrospective evaluation, there may have been a bias towards undocumented bleeding complications [5].

### Acquired bleeding disorders

There are several acquired diseases that can directly affect the normal hemostasis. These include, for example, pathologies of the liver, kidneys, bone marrow and/or immune system.

### Liver diseases

It is known that patients with (severe) liver diseases have altered blood coagulation of a multifactorial etiology. Most coagulation factors as well as thrombopoietin are formed in the liver. The progression of liver diseases is associated with a decrease in platelet adhesion to the endothelium along with lower platelet aggregation and poorer platelet activation [29]. Portal hypertension also leads to hypertrophy of the spleen and an inverse relationship between spleen size and platelet count via sequestration of platelets within the spleen. Thus, thrombocytopenia represents the most common hematologic complication in the presence of chronic liver diseases [30, 31].

Furthermore, quite a few of the affected patients exhibit chronic anemia, depletion of vitamin K due to malnutrition, decreased intestinal absorption or impaired hepatic storage function, increased fibrinolytic activity, and even, especially in the case of ethyltoxic liver injury, additional bone marrow suppression [32]. Both prothrombin time (PT) and activated partial thromboplastin time (aPTT) are prolonged in chronic liver disease and severe bleeding can occur after dental implantation [33]. For this reason, it is recommended that liver function and coagulation tests are performed in affected patients prior to invasive oral procedures.

Patients at risk of vitamin K malabsorption may require oral or intravenous vitamin K substitution before the procedure, and patients with advanced liver damage may require additional pre-interventional plasma or platelet infusion [34]. Despite advances in collection, storage and transfusion of platelet concentrate, there are risks such as infections, alloimmunization, as well as febrile and non-hemolytic reactions [35]. Local hemostyptic measures should always be applied because prophylactic transfusions alone do not result in adequate hemostasis despite apparently “normal” values [36]. Overall, it is important to contact the patient’s primary care physician/hematologist regarding patient preparation to quantify the expected interventional risk [37–39].

Some authors were able to identify a significant association between liver cirrhosis and periodontitis [40–42]. In final liver disease and before transplantation, rehabilitation of dental foci is the therapeutic gold standard [43]. Accordingly, Concero et al. performed 1329 tooth extractions in 346 patients before liver transplantation [44]. Patients with platelet count of < 50,000/μL (this threshold is generally used although it is based on expert opinion and may expose patients to unnecessary transfusions [45]) received a platelet concentrate, and in patients with an International Normalized Ratio (INR) > 2.5, procedures were postponed until lower values could be achieved. Local hemostyptic measures were performed (curettage, digital pressure, insertion of gelatin sponges, adaptive suturing, compresses soaked in tranexamic acid). Bleeding events were recorded in 1.4% of the cases [44]. Accordingly, Pereira et al. reported rebleeding in 1.7% of tooth extractions in liver transplant candidates [32]; in other studies, the rebleeding rate was 0–2.9% [34, 36, 46]. In contrast to Cocero et al., Medina et al. concluded that patients with liver cirrhosis do not require transfusion products above a platelet count of 16,000/μL and an INR < 3 and that the use of local hemostatic measures is sufficient in these cases [47]. The comparison of preoperative intranasal desmopressin administration with preoperative plasma transfusion in individuals with a platelet count of 30–50,000/μL and INR of 2–3—both in combination with local hemostatic measures—showed no differences regarding postoperative bleeding events [48]. In contrast, extractions in patients with undiagnosed liver disease and without appropriate precautions may quite well result in life-threatening bleeding [49, 50].

*Conclusion* In patients with liver disease prior to dental surgical procedures, preoperative preparation is essential and prophylactic transfusion measures alone do not ensure adequate hemostasis. Patients with severe liver disease could safely undergo oral surgical procedures when receiving prophylactic transfusions combined with (local) hemostatic measures and close monitoring.

### Renal diseases

Renal diseases result in impaired hematopoiesis for several reasons: Among other factors, platelet production is restricted due to compromised thrombopoietin secretion [51]; intrinsic platelet defects occur, as healthy renal glomerular endothelial cells express vWF, abnormal platelet adhesion is present in renal injury and general vasodilatation occurs due to increasing prostaglandin levels [37]. Erythropoietin is also impaired, potentially leading to anemia. This anemia increases the uremic tendency of qualitative thrombocyte dysfunction. In uremic patients, this disorder can be controlled, for example, by administration of Desmopressin, conjugated estrogen, erythropoietin, dialysis or by infusions of platelet concentrates [52].

In case of advanced kidney disease, dialysis is the therapy of choice. Hemodialysis requires the use of an anticoagulant in the form of heparin to maintain access patency and facilitate filtration of toxic blood compounds. This heparinization associated with mechanical trauma to platelets may decrease platelet counts and increase hemorrhagic risk. This tendency is exacerbated by preexisting capillary fragility and anemia. Particularly, special attention should be paid to the effect of heparin in patients with chronic renal failure undergoing hemodialysis, before and during oral surgical treatment [53].

Communication between the dentist and nephrologist is highly recommended in these cases. Dental treatment with the risk of bleeding should be postponed to the day without dialysis [54]. In an emergency, Protamine can be administered as an antagonist to heparin. For patients receiving oral anticoagulation with vitamin K antagonists, the INR should be determined before intervention, although minor surgical procedures can be safely performed with an INR < 4 [55, 56]. Pendem et al. performed tooth extractions in 36 patients with severe renal disease using antihypertensive and hemostatic measures (local anesthesia without epinephrine, socket curettage, gelatin sponges, oxidized cellulose, and sutures) without postoperative bleeding events [57]. Overall, local hemostyptic measures should always be used [38, 54]. Greenwood et al. additionally recommend the (intranasal) administration of Desmopressin to prevent postoperative bleeding [37]. Because of renal excretion of numerous pharmaceuticals, renal dysfunction may lead to an increased bleeding tendency associated with oral anticoagulants/antiplatelet agents [58].

*Conclusion* When treating patients suffering from kidney disease, prior to dental surgical procedures, it is important to closely coordinate the planned procedure with the nephrologist in charge to consider a possible deterioration of the patient’s general condition during and after the dental treatment.

Hemostasis protocols are based on the severity of the disease and the planned procedure. In addition to bleeding tendencies, special attention should be paid to drug interactions.

### Bone marrow diseases

The bone marrow produces hematopoietic stem cells and normally only mature cells are released from the bone into the systemic circulation. Any disorder that causes an abnormality in the production of immature progenitor or mature cells may alter the bleeding tendency of affected patients. Normal bone marrow function can also be disrupted by infections such as tuberculosis or malignancies like leukemia [38].

Leukemia is a heterogeneous group of hematologic disorders characterized by increased and uncontrolled production of nonfunctional leukocytes. By spreading in the bone marrow, they displace the usual hematopoiesis, resulting in anemia, thrombocytopenia and leukopenia. In the case of acute myeloid leukemia, Mester et al. only recommended invasive dental procedures in the presence of a platelet count of > 50,000/μL and a neutrophil count of > 100/μL [59]. In a retrospective cohort study including 68 patients with a total of 200 dental extractions, an increased bleeding tendency was reported when platelet counts were 20,000/μL [60]. Another guideline states that although prophylactic platelet concentration is not required before dental extractions, transfusions with the goal of a platelet count of > 10,000 may be useful [61]. In summary, in the case of thrombocytopenia, thrombocyte transfusions may be necessary before invasive dental procedures [38]. If the patient is already receiving systemic therapy, invasive dental procedures are appropriate during periods of remission or between chemotherapy cycles while cell and platelet counts are at their optimum. Accordingly, Akashi et al. did not detect any rebleeding events when platelet counts were > 50,000/μL and topical hemostyptics were used [62].

*Conclusion* Consultation of the treating general physician / hematologist and the application of local hemostyptic measures including the use of Desmopressin and tranexamic acid is of paramount importance.

### Oral anticoagulants and antiplatelet therapy

**Fig. 5:**
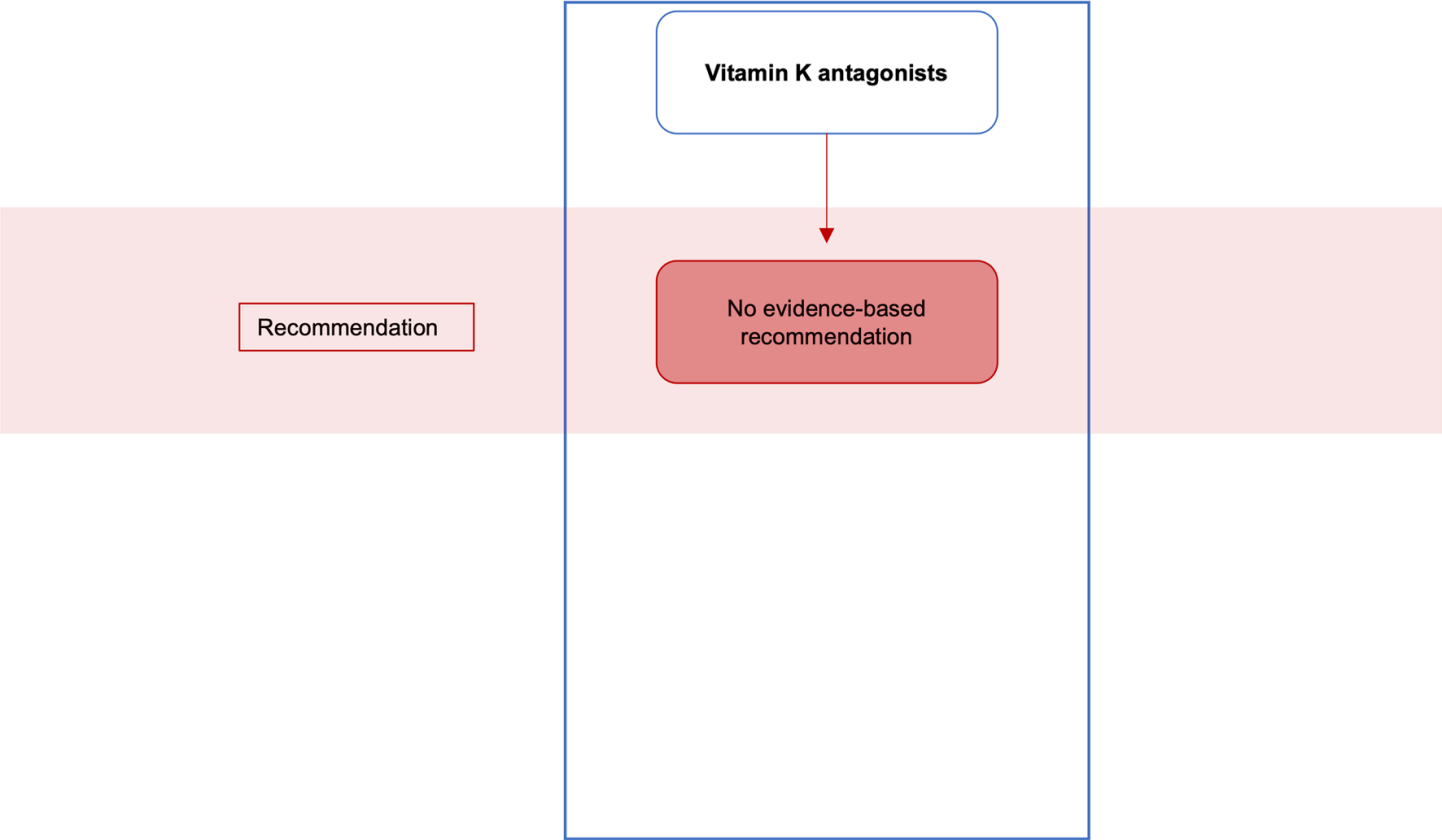
Clinical recommendations for oral surgery in patients treated with vitamin K antagonists

**Fig. 6:**
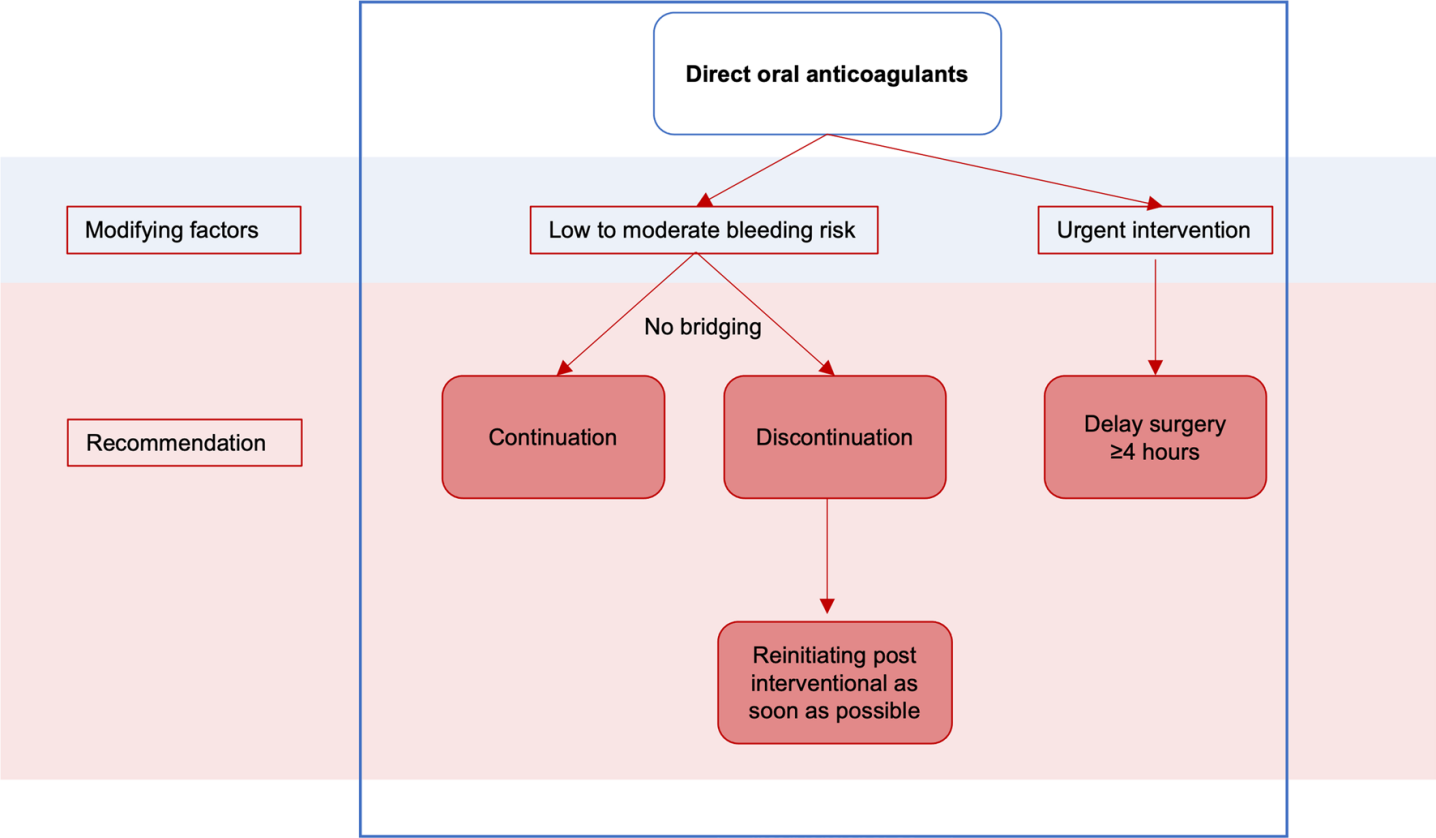
Clinical recommendations for oral surgery in patients treated with direct oral anticoagulants

**Fig. 7:**
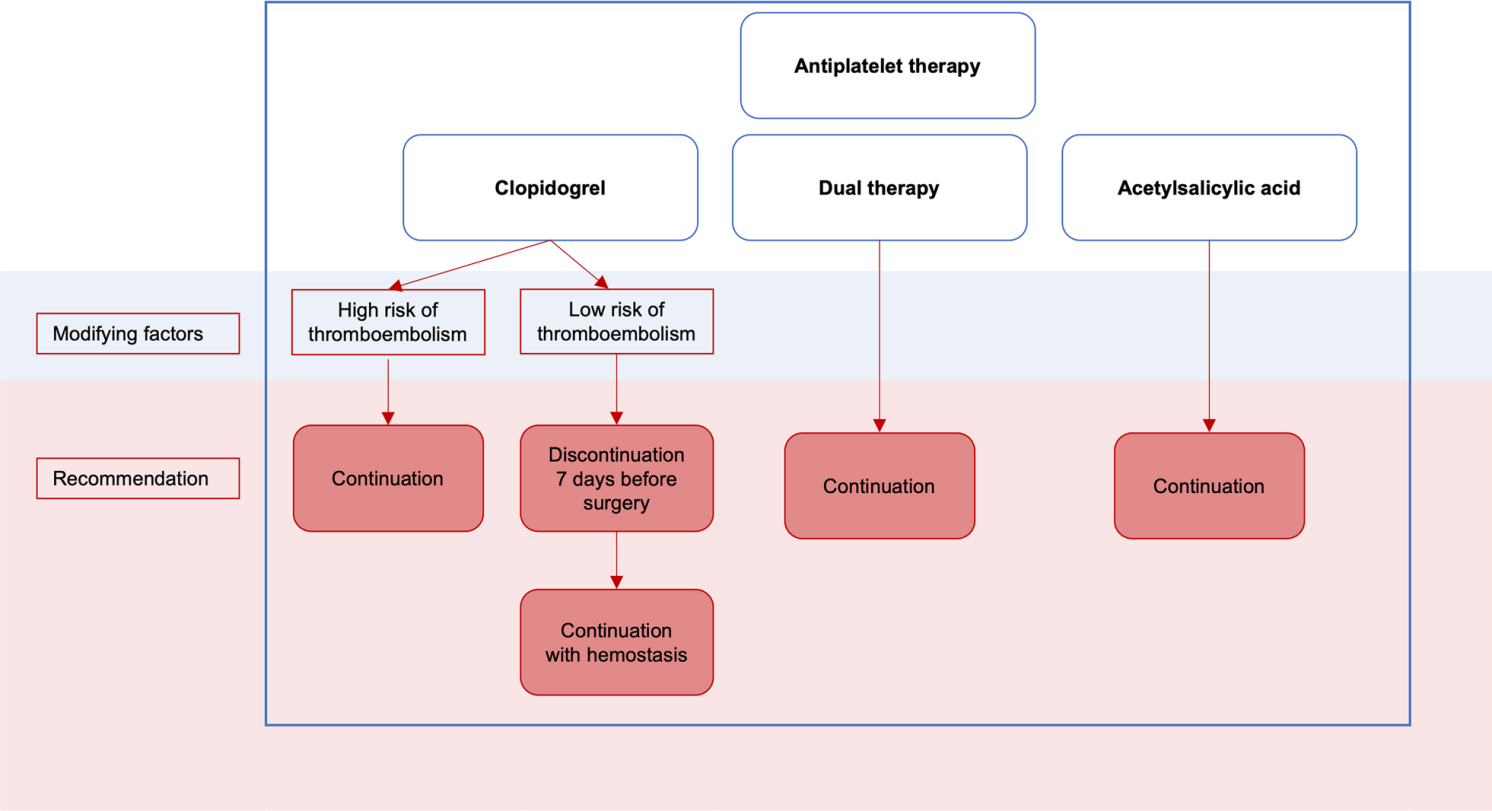
Clinical recommendations for oral surgery in patients treated with antiplatelet therapy

Approximately one percent of the German population is treated with oral anticoagulants. When a patient treated with oral anticoagulants must undergo an elective procedure or a comparatively minor operation such as implant placement, the risk of bleeding must be weighed against the risk of thrombosis associated with interruption of anticoagulant medication [4, 63]. According to Nizarali et al., dental procedures that are unlikely to cause bleeding include routine conservative, restorative, and orthograde endodontic procedures with buccal, intraligamentary, and intrapapillary local anesthesia, supragingival scaling, prosthodontic procedures, and adjustment of orthodontic appliances. In these cases, implementation without measuring INR, discontinuing heparin therapy, or changing the anticoagulant medication is recommended. However, the scenario is different for other surgical procedures and types of local anesthesia with a higher bleeding risk [38, 53]. Recommendations for oral surgery in patients treated with oral anticoagulants and antiplatelet therapy are displayed in Figs. 5, 6, 7.

### Anticoagulation with vitamin K antagonists

Vitamin K antagonists inhibit the carboxylation of the vitamin K dependent coagulation factors II, VII, IX and X, as well as proteins C and S. Therapeutic levels of vitamin K antagonists are monitored by measuring the INR, which should be kept within a narrow therapeutic range to prevent thromboembolic events without causing bleeding complications. After determination of the individual risk pattern, patients can be assigned to either a low-intensity (2.0–3.0) or high-intensity (2.5–3.5) INR therapy range [64].

### Anticoagulation with direct oral anticoagulants

Direct Oral Anticoagulants (DOACs) were developed as an alternative to vitamin K antagonists. Their main advantages include an immediate onset of action, fewer drug and food interactions, a short half-life, and fixed-dose anticoagulation without the need for regular monitoring. DOACs act by directly inhibiting activated clotting factors. Currently available DOAC include Dabigatran (inhibiting factor IIA) and rivaroxaban, edoxaban and apixaban (each inhibiting factor Xa) [65]. Based on cumulative evidence from large randomized multicenter trials, DOACs have been shown to be noninferior to vitamin K antagonists in preventing thromboembolic events, although the risk of serious bleeding events may be lower in patients with atrial fibrillation [66, 67]. In older studies, the death rate of patients treated with DOACs who experienced severe bleeding was similar to or even lower compared to patients treated with vitamin K antagonists, despite the lack of availability of antidotes [68]. Two antidotes were approved: idarucizumab to dabigatran and andexanet alfa to rivaroxaban / apixaban reversal [69, 70]. However, in addition to the high cost of antidotes, it should be noted that the incidence of thromboembolic events increased dramatically to as high as 18% after administration of the approved antidotes [71].

### Antiplatelet therapy

Antiplatelet agents are prescribed for primary and secondary prevention of cardiovascular disease, treatment of acute myocardial and cerebral ischemia, and long-term treatment of (transient) ischemic stroke. They are used alone or in combination with other antiplatelet agents or anticoagulants and inhibit platelet aggregation and thrombus formation. Acetylsalicylic acid exerts its anticoagulant effect even at low doses via irreversible inhibition of cyclooxygenase-1 (COX-1), thus preventing thromboxane A_2_ formation and having an effective duration analogous to platelet survival (8–11 days). clopidogrel, ticlopidine, prasugrel and ticagrelor inhibit the adenosine phosphate (ADP)-induced expression of GP IIb/IIIa via inhibition of the P2Y2 receptor, which is required for platelets to attach to fibrinogen and other platelets.

### A treatment tool for patients with anticoagulant or antiplatelet therapy

The patient and the physician have four options before invasive procedures:

1. Discontinuation of medical therapy before and after the procedure
2. Continuation of the therapy
3. Reduction of anticoagulant/antiaggregational therapy
4. Temporary discontinuation of oral anticoagulant therapy while providing a comparatively short-acting anticoagulant (e.g., unfractionated or low-molecular-weight heparin) to bridge the perioperative period.

### Oral anticoagulants—thromboembolism risk by discontinuing the medication

The risk of thromboembolism when discontinuing vitamin K antagonists has been poorly studied. This represents a critical gap in current knowledge because the bleeding risk associated with “bridging” by heparin is also justified by the fact that such bridging therapy prevents potentially lethal thromboembolic events that would otherwise occur. Without sufficient knowledge concerning the individual risk of potential thromboembolism events, an appropriate risk–benefit evaluation cannot be adequately performed, however, some publications repeatedly cited refer on unclear and/or poorly described events [72, 73].

In a first prospective multicenter cohort study, Garcia et al. reported of 1024 patients anticoagulated with Warfarin in whom the anticoagulant had been bridged with low-molecular-weight heparin in 8.4% of the cases and discontinued in the remainder of cases. In 83.8% of the cases, the duration of anticoagulant-discontinuation was ≤ 5 days. During the 30-day follow-up period, seven patients in whom the anticoagulant had been discontinued and not bridged developed thromboembolic events. Four of the patients with thromboembolic events had primarily received Warfarin because of atrial fibrillation. Furthermore, in this group, 9 secondary bleeding events (0.8%) occurred. In the group with bridging 14 events of secondary bleeding (13%) emerged. Overall, the study showed that thromboembolic events are rare but do occur in low- to intermediate risk outpatients undergoing short-term minor intervention if Warfarin therapy is interrupted. On the other hand, analogous to the literature, “bridging” with Heparin was found to increase the risk of bleeding significantly [74–76]. Despite the large number of patients and cases in the study by Garcia et al., the small number of thromboembolic events [which, is similar to rates with interrupted administration as well as continued administration of DOACs and vitamin K antagonists (0.16–1.9%)] [77–81] limits the ability to draw definitive conclusions about the overall risk of interrupting perioperative therapy with a vitamin K antagonist for each individual patient. The risk of peri-interventional thromboembolism is certainly influenced by several patient-, procedure-, and drug-specific factors that collectively influence thrombogenicity. Furthermore, Warfarin has a significantly shorter half-life period than phenprocoumon, which is commonly used in Germany and thus cannot be directly compared.

### Oral anticoagulants—bridging or no bridging?

Low-molecular-weight heparin is often recommended as part of so-called “bridging” during temporary interruption of oral anticoagulants, although its safety and efficacy has not been proven for all procedures and for all patient-specific risk groups. In 2012, Siegal et al. concluded in their systematic literature review that patients who received “bridging”, compared with a group in which oral vitamin K antagonist therapy was continued, had a higher bleeding risk and a similar rate of thromboembolic events [79, 82]. In the following years, an increasing number of studies demonstrated the practical advantages of continued therapy with oral anticoagulants over perioperative bridging [83]. In 2015, Douketis et al. addressed the question of whether peri-interventional bridging with low-molecular-weight heparin is necessary in patients with atrial fibrillation and existing oral anticoagulation with Warfarin. For this purpose, they conducted a randomized, double-blinded, placebo-controlled clinical trial comparing 891 bridged patients with 913 patients receiving a placebo regarding bleeding and thromboembolic events in minor (89%) and major surgical procedures at a follow-up duration of 30 days. In total, there was no apparent difference in terms of thromboembolic events between the groups. Bleeding events occurred significantly more frequently in the bridging group [80]. In 2017, Young et al. published a systematic review comparing the risk of major bleeding and thromboembolic events in patients bridged with heparin versus patients with continued oral anticoagulation (vitamin K/DOAK). Here, bridging led significantly more often to bleeding events (odds ratio [OR]: relevant bleeding 3.23; OR minor bleeding 1.52). Between the two groups, no differences in thromboembolic events were observed [84].

DOACs, with their short half-life period (5–15 h in patients with normal renal function), were shown to have a low incidence of thromboembolic events (0.2–0.6%) without bridging, whereas bridging with heparin resulted in an increase in bleeding complications without additional thromboembolic benefit. Thus, continuation or short-term interruption of DOAC therapy without bridging has been recommended for most invasive procedures with low to moderate bleeding risk [80, 85].

### Oral anticoagulants—discontinue or continue?

In a systematic review by Kämmerer et al., it was concluded that dental surgical procedures such as implant insertion in patients under oral anticoagulation with vitamin K antagonists can be safely performed without changing the anticoagulant regimen if the anticoagulation is in the therapeutic range (INR < 4) and local hemostatic measures are implemented [86]. Other studies reported similar results [87, 88]—incorporating a study with DOAC [89]. Hiroshi et al. conducted a multicenter, observational cross-sectional study, examining data from 496 patients in which vitamin K antagonization with Warfarin had been continued for tooth extraction (INR ≤ 3, 7 days before intervention) and compared them with 2321 patients treated while vitamin K antagonist therapy was discontinued. Bleeding events were significantly more frequent in the group receiving ongoing anticoagulant therapy, whereby age < 65 years, higher pretherapeutic INR, additional use of antiplatelet agents, and the presence of current inflammation at the extraction site positively correlated with those very bleeding events [90]. In contrast, a meta-analysis by Yang et al. was able to calculate a non-significantly different incidence of postoperative bleeding complications of 10.8% with continued oral anticoagulation versus 8.3% with discontinuation of anticoagulation for tooth extraction. In every included study, no severe bleeding occurred while using local hemostatic measures and sufficient hemostasis could be achieved in all cases by application of local measures [91].

Hanken et al. compared 52 dental surgical procedures (osteotomy, implantation) under Rivaroxaban (20 mg/days) with 285 procedures without anticoagulation in a retrospective observational study. The authors found a significantly higher bleeding rate (11.5% versus 0.7%) in the DOAC patient group [92]. In contrast, no difference could be observed in other studies [44, 93, 94]. Galletti et al. placed 57 implants in 12 patients receiving Rivaroxaban (discontinued 24 h before the procedure) without postoperative bleeding events [65]. In a recent literature research including six studies, Manfredi et al. were also unable to identify any differences in bleeding and thromboembolism in patients undergoing invasive dental procedures, while DOAC was either continued or (briefly) discontinued [90].

Mauprivez et al. compared 31 patients on continued DOAC medication with 20 patients on continued vitamin K antagonization (INR 2–3) for tooth extraction while using obligatory hemostatic measures and found no differences in terms of bleeding events. Notably, all bleeding events in the factor Xa inhibitor group occurred in patients in whom the intervention took place < 4 h after the last dose of DOAC [95].

Therefore, in the case of urgent interventions requiring surgery within hours, delaying surgery by at least 4 h (pharmacologically reasonable 12–24 h) after last DOAC administration should be considered, because a substantial amount of the drug is eliminated within this period of time [70, 96]. Overall, as discussed above, bridging is currently not recommended with DOAC, although either short discontinuation or continuation of DOAC appears to be appropriate for interventions with low bleeding risk such as dental surgical procedures. If DOAC is discontinued, it should be reinitiated post-interventional as soon as possible [97]. Kim et al. subsumed the current evidence (Table 2), with dental surgical procedures most likely to be classified in the minimal to lower bleeding risk group [82].

**Table 2:** Perioperative management of patients taking DOAK during elective interventions (based on [101])

Duration of DOAC interruption				
	*Dabigatran*		*Rivaroxaban, apixaban, edoxaban*	
	Surgical bleeding risk		Surgical bleeding risk	
Creatinine-clearance	Low	High	Low	High
	Recommended time of DOAC interruption before surgery		Recommended time of DOAC interruption before surgery	
≥ 80 ml min	≥ 24 h	≥ 48 h	≥ 24 h	≥ 48 h
50–79 ml/min	≥ 36 h	≥ 72 h	≥ 24 h	≥ 48 h
30–49 ml/min	≥ 48 h	≥ 96 h	≥ 24 h	≥ 48 h
< 30 ml/min	No sufficient evidence		≥ 36 h	≥ 48 h

### Antiplatelet therapy—discontinue or continue?

Analogous to oral anticoagulation, there are no reports of uncontrollable or even lethal bleeding events under antiplatelet therapy during invasive dental procedures. For dentoalveolar surgery, several smaller prospective observational studies investigated potential bleeding complications while continuing acetylsalicylic acid. They concluded no significant differences in intraoperative blood loss, bleeding duration, or bleeding complications when local hemostatic measures were used [98–100]. A meta-analysis including 14,981 patients in whom perioperative continuation was compared with discontinuation of low-dose acetylsalicylic acid found that 0.6% of patients with discontinued acetylsalicylic acid therapy suffered vascular events [101]. In addition, a meta-analysis of 50,279 patients showed that discontinuation of acetylsalicylic acid had adverse effects. It was correlated with a threefold increased risk of serious cardiac events, with an even increased risk (OR 89.78) for patients with intracoronary stents [102].

Monotherapy with Clopidogrel should be continued for dental surgical procedures [103], but—according to expert consensus—can also be discontinued seven days before surgery in patients at low risk of thromboembolism and continued after surgery once hemostasis is achieved [104, 105].

Discontinuation of dual therapy with acetylsalicylic acid and Clopidogrel is associated with a 5- to 10-fold increased risk of myocardial infarction in patients with coronary stents. Here, the risk appears to be inversely proportional to the date of prior cardiac intervention [106]. In contrast, in studies analyzing patients receiving dual antiplatelet aggregation only minor bleeding could be reported in the setting of dental surgical procedures that could be stopped by using local hemostatic measures [107, 108].

### Hemostatic measures in patients with anticoagulant or antiplatelet therapy

For most patients receiving oral anticoagulants and/or antiplatelet agents, local compression is sufficient to active hemostasis [109, 110], although, in particular, an increased INR, difficulty in achieving hemostasis intraoperatively, and higher serum creatinine concentrations may be additional risk factors for postoperative bleeding [111]. If further measures are necessary, the use of sutures, collagen, oxidized cellulose, absorbable sponges, chitosan, bone wax, and fibrin glue, for example, have proven to be effective [112–114] and can be used to achieve successful hemostasis even with INR values outside the therapeutic range [115]. However, evidence as to which hemostatic regimen should be preferred cannot be extracted from the literature [86, 109, 112].

In a systematic Cochrane literature review, Engelen et al. evaluated the efficacy of antifibrinolytics in preventing bleeding complications in patients receiving oral anticoagulation (vitamin K antagonists or DOACs) undergoing dental surgery. Overall, a positive effect of locally applied tranexamic acid (mostly 5% concentration, 3–4 × /days) to prevent oral bleeding events could be demonstrated [116].

## Conclusion

Despite the limited evidence, a treatment guide could be developed by means of this narrative review to improve safety for patients and practitioners. The major shortcoming of this study is the lack of a systematic approach to this review. Prospective randomized controlled trials are needed to allow the implementation of official evidence-based guidelines.

## Data Availability

The data that support the findings of this study are available in the National Center for Biotechnology Information at https://www.ncbi.nlm.nih.gov/.

## Abbreviations

RCT: Randomized controlled trial
VWD: Von Willebrand disease
VWF: Von Willebrand factor
NSAID: Non-steroidal anti-inflammatory drugs
DeBRATT: Dental Bleeding Risk Assessment and Treatment Tool
PT: Prothrombin time
aPTT: Activated partial thromboplastin time
INR: International normalized ratio
DOAC: Direct oral anticoagulant
COX-1: Cyclooxygenase-1
ADP: Adenosine phosphate
OR: Odds ratio
